# The histone methyltransferase NSD3 oncogene triggers ribosomal DNA transcription, interfering with FOSL2 in cancer

**DOI:** 10.1038/s41419-026-08768-0

**Published:** 2026-05-04

**Authors:** Federica Corigliano, Marco Gaviraghi, Alessio Lupi, Antonino Alex Cartalemi, Guido Gatti, Simona Punzi, Gemma Crupi, Oronza A. Botrugno, Francesca Bernassola, Elena Torlai Triglia, Aurora Negro, Francesco Ghini, Giovanna Musco, Davide Cittaro, Jose M. Garcia-Manteiga, Simona Segalla, Giovanni Tonon

**Affiliations:** 1 Functional Genomics of Cancer Unit, Comprehensive Cancer Center, IRCCS San Raffaele Scientific Institute, Milan, Italy; 2 Department of Experimental Medicine, TOR, University of Rome Tor Vergata, Rome, Italy; 3 Università Vita-Salute San Raffaele, Milan, Italy; 4 Biomolecular Nuclear Magnetic Resonance Laboratory, Division of Genetics and Cell Biology, IRCCS San Raffaele Scientific Institute, Milan, Italy; 5 Center for Omics Sciences, IRCCS San Raffaele Scientific Institute, Milan, Italy

**Keywords:** Oncogenes, Histone post-translational modifications, RNA, Transcriptional regulatory elements

## Abstract

Ribosome biogenesis is pervasively enhanced in cancer, and yet, how the expression of ribosomal RNA genes is triggered remains largely unknown. We found that the long isoform of the histone methyltransferase NSD3, NSD3L, one of the most frequently amplified genes in cancer, reshapes the chromatin environment surrounding ribosomal DNA (rDNA), thus triggering rRNA expression. An unbiased mass-spec approach revealed that NSD3L binds several nucleolar proteins and localizes to the nucleolus. NSD3L is essential for the binding of Polymerase I as well as of its activator UBTF (Upstream Binding Transcription Factor) to the entire rDNA locus. Conversely, NSD3L binds to a narrow sequence on rDNA located upstream of the rRNA transcription start site (TSS), displacing FOSL2, a member of the FOS/JUN transcription factor family. Upon NSD3L ablation, FOSL2 increases its binding to this region, leading to reduced rDNA expression. These results suggest a surprising, repressive role for FOSL2 on ribosomal gene expression. We also determined that NSD3L competes with another suppressor of rDNA expression, the histone methyltransferase SUV4-20H, thus impeding the deposition of the repressive histone mark H4K20me3 on rDNA. Therefore, NSD3L balances and counteracts the activities of FOSL2 and SUV4-20H on rDNA, unleashing rDNA synthesis. Accordingly, NSD3L overexpression is associated with enhanced expression of nucleolar genes in several tumor types. We hence propose NSD3L as a central epigenetic orchestrator of rRNA transcription in cancer and an enticing therapeutic target for the large group of cancers presenting with NSD3 amplifications and overexpression.

## Introduction

Ribosome biogenesis is a fundamental and tightly regulated process that controls cell growth and proliferation. Ribosomal RNAs (rRNAs) are key constituents of the two eukaryotic ribosomal subunits 40S and 60S. In a typical diploid human genome, there are approximately 400 ribosomal DNA (rDNA) copies [1, 2] encoding for rRNAs. These rDNA units are arranged in tandem repeats, separated by intergenic spacer sequences (IGS), and are located within the short arm of five acrocentric chromosomes. The nucleolus, a prominent non-membrane-bound organelle [3], forms around clusters of repeated rDNA genes and serves as the site of rRNA synthesis and early ribosome assembly [4].

A dedicated polymerase, RNA polymerase I (Pol I), drives transcription of rDNA, generating the 47S rRNA precursor rRNA that is processed into 18S, 5.8S, and 28S rRNAs. The localization of Pol I to rDNA is mediated by a series of protein-protein interactions. Pol I recruitment to rDNA is centered on the upstream binding factor (UBTF) [5]. UBTF, Pol I-specific transcription factor, contributes to the formation of the pre-initiation complex (PIC), recruits the selectivity factor 1 (SL1) to rDNA, and guides transcription initiation and elongation [6]. Conversely, loss of the association of UBTF with rDNA genes promotes the switch from active to silent nucleolar organizing regions (NORs), contributing to rDNA copy inactivation and nucleolar reorganization [7, 8].

Cancer cells consistently present increased rDNA transcription and ribosome biogenesis to support uncontrolled growth, and pharmacological inhibition of Pol I–dependent transcription is being actively explored as an anticancer strategy [3, 9, 10].

What controls rDNA transcription? Several studies have explored the chromatin landscape surrounding rDNA. Active rDNA units are enriched in acetylated histones H3 and H4, as well as H3K4me3, whereas inactive rDNA promoters are marked by repressive histone modifications, including H3K9me, H4K20me, and H3K27me [11, 12]. Several chromatin-associated complexes that repress rDNA transcription have been identified, such as NoRC, eNoSC, and NuRD [13], along with specific histone methyltransferases, including SETDB1, SUV39H1, and SUV420H2 [14], and lysine demethylases, including KDM2A, JHDM1B, KDM4A [15] and JHDM1B [16]. However, limited knowledge is available on factors or complexes able to increase rRNA expression. The only exception is the oncogene MYC that directly binds to the promoters of Pol I-specific transcription factors, like UBTF and SL1, eliciting their transcription [17, 18].

The nuclear receptor–binding SET domain NSD protein family of methyltransferases – comprising NSD1, NSD2, and NSD3 (also known as Wolf-Hirshhorn syndrome candidate 1 like 1 (WHSC1L1) – plays important roles in development and disease. These proteins share several domains, including an HMGB, two PWWP, and four zinc finger domains [19], alongside the catalytic SET domain responsible for methylation of histone H3 on lysine 36 (H3K36) [20]. The oncogenic role and mechanisms by which NSD1 and NSD2 are tumorigenic have been extensively explored, while how NSD3 triggers carcinogenesis remains largely unknown [21, 22].

NSD3 is located on chromosome 8p12, a region frequently subject to focal amplification in human cancer. The 8p12-p11.2 amplification, in which NSD3 represents the main target gene, is the most frequent genetic lesion in squamous cell lung cancer [23–25] and is also observed in breast, pancreatic, colorectal, and head and neck cancers, where it correlates with poor prognosis [23, 26–28]. In addition, highly oncogenic fusion proteins involving NSD3, namely NSD3-NUP98 and NSD3-NUT, have been identified in acute myeloid leukemia, NUT midline carcinoma, and thyroid carcinoma [29–31].

In human cells, alternative splicing gives rise to two NSD3 transcripts: a short isoform (NSD3S, 645 amino acids), which contains an N-terminal PWWP domain that lacks the catalytic SET domain, the less characterized WHISTE isoform, and a long isoform (NSD3L, 1,437 amino acids) that includes all the domains, comprising the SET methyltransferase domain [32]. Most studies addressing NSD3's role in cancer have focused on NSD3S, demonstrating its oncogenic role in breast cancer, where it promotes ESR1/ERα over-expression [33], and in acute myeloid leukemia, where it acts as an adaptor linking BRD4 to the chromatin remodeling protein CHD8 [34, 35]. By contrast, a few studies on NSD3L showed a role in promoting G1-S phase transition in head and neck squamous cell carcinoma, mediated by the H3K36 methylation of CDC6 and CDK2 cell cycle genes [28]. Moreover, NSD3L can methylate non-histonic substrates, including EGFR, enhancing its oncogenic activity [36]. Importantly, consistent with a critical role for its catalytic function, expression of a hyperactive mutant SET domain of NSD3L triggers squamous lung cancer development and progression in vivo [24].

In this study, we identified NSD3L as a key player in eliciting ribosomal RNA transcription in cancer cells. We determined that NSD3L resides in nucleoli, where it interacts with nucleolar proteins, focally binds the rDNA locus, enhancing rRNA transcription. NSD3L remolds the chromatin environment on ribosomal loci, thus favoring the binding of Pol I and UBTF to rDNA while preventing a surprising repressive role of FOSL2 and of SUV4-20H on ribosomal RNA expression.

## Results

### NSD3L interacts with nucleolar proteins

**Fig. 1:**
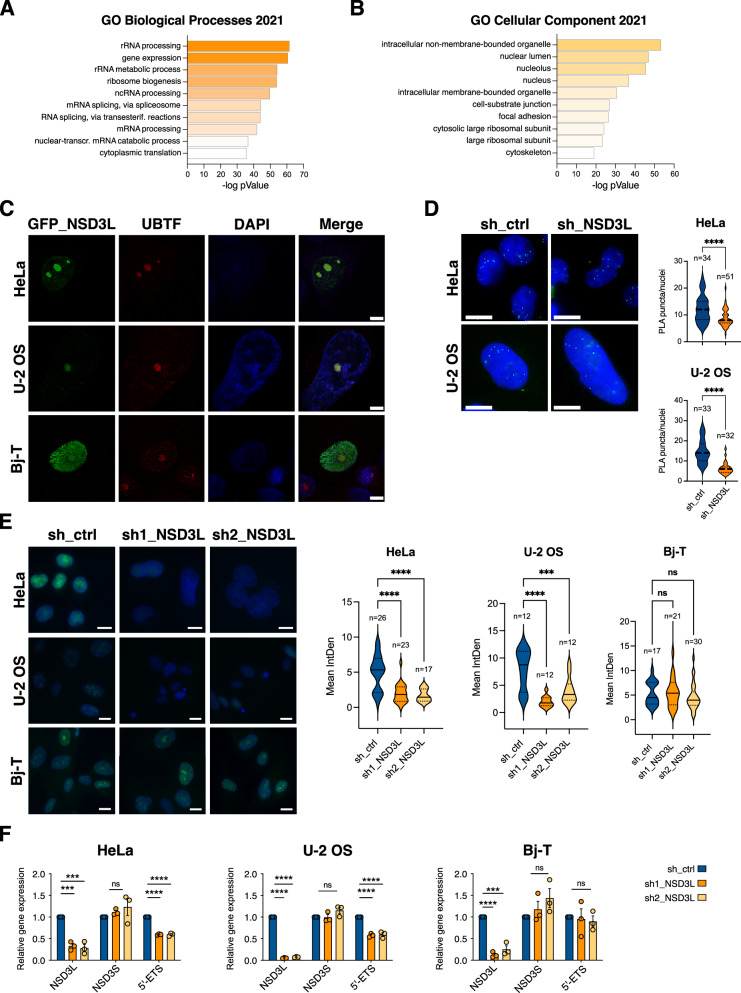
NSD3L interacts with nucleolar proteins and localizes in the nucleolus. **A** Top 10 enriched pathways using GO Biological Processes 2021 or **B** Cellular Component 2021 using Enrichr for NSD3L interactors identified with mass spectrometry. **C** Confocal microscopy on HeLa, U-2 OS, and Bj-T cells, expressing the N-terminal GFP-NSD3L fusion protein and stained with an anti-UBTF nucleolar marker. Scale bar: 7 µm. **D** Proximity Ligation Assay (PLA) foci quantified as PLA puncta/nuclei in HeLa and U-2OS cells in sh_ctrl and sh_NSD3L conditions. Significant differences among groups were quantified by applying Student’s t-test (****p < 0.0001). Scale bar: 10 µm. **E** Representative images showing EU incorporation followed by Click-iT® assay (scale bar: 10 µm) on the left. On the right, quantification of EU signal intensity expressed as mean integrated intensity (IntDen). Data are represented as mean (± SD) of three independent biological replicates. One-way ANOVA test was performed, with multiple comparisons (***= *p* < 0.001, ****= *p* < 0.0001). **F** RNA expression levels of NSD3L, NSD3S, and 5’ ETS, assessed with Real Time qPCR on HeLa, U-2 OS, and BJ-T cell lines, silenced with two different shRNAs targeting NSD3L. Data are normalized first on GAPDH, then to the control condition. Results are represented as mean (± SD) of three independent biological replicates. Where indicated, a two-tailed Student’s *t*-test was performed (*** *p* < 0.001, ****p < 0.0001).

To shed light on the function of the NSD3 long isoform, we sought to define the NSD3L interactome with an unbiased proteomic approach. To this end, HeLa cells, which express low levels of NSD3L (Fig. S1A, B), were transiently transfected with a GFP-tagged NSD3L-expressing vector and the lysates subjected to protein immunoprecipitation (IP). The overexpression attained was comparable with the levels present in cancer cell lines amplified for NSD3 (Fig. S1A, B). After the evaluation of IP efficiency through Western Blotting (WB, Fig. S1C), IPs were analyzed by mass spectrometry (Fig. S1D). This analysis revealed 346 proteins (Supplementary Table S1) as potential NSD3L interactors. To determine whether the identified putative interactors belonged to specific pathways or cellular districts, we performed pathway enrichment analysis [37]. Pathways engaged in rRNA processing and ribosome biogenesis were the most prominently enriched (Fig. 1A and S2A, B). Accordingly, the nucleolus was among the most significantly enriched cellular localizations of NSD3L interactors (Fig. 1B, S2C). Immunoprecipitation with endogenous NSD3, using a specific antibody, in both HeLa (Fig. S1E) and U-2 OS osteosarcoma (Fig. S1F) cell lines confirmed the interaction of NSD3L with several nucleolar proteins. Importantly, we tested and excluded potential interactions of NSD3S with nucleolar proteins (Fig. S1G).

### NSD3L resides within nucleoli

Prompted by the mass spectrometry results, we hypothesized that NSD3L may localize in the nucleolus. To this end, we transiently transfected HeLa and U-2 OS cells with a fusion GFP-NSD3L protein and assessed NSD3L localization with confocal microscopy. This analysis revealed that NSD3L co-localized with the nucleolar marker UBTF in both cell lines (Fig. 1C, upper and middle panel and Fig. S1H), thus suggesting that NSD3L resides within nucleoli. To assess whether the nucleolar localization of NSD3L was specific for cancer cell lines, we also transiently transfected BJ-T, an immortalized non-transformed cell line, with GFP-NSD3L. NSD3L presented a diffused signal in the nucleus, with only a weak staining signal in the nucleolus (Fig. 1C, lower panel). Of note, we excluded NSD3S localization in the nucleolus (Fig. S1J).

We confirmed that NSD3L resides in nucleoli with a proximity ligation assay (PLA), where we probed the potential nucleolar co-localization of endogenous NSD3 and UBTF. We detected several PLA spots both in HeLa and U-2 OS cell lines, supporting the close interaction between NSD3 and the nucleolar protein UBTF (Fig. 1D, left panel). As expected, NSD3L knockdown reduced the number of PLA dots (Fig. 1D right panel and Fig. S1I). These data further support the nucleolar localization of NSD3 in cancer cells.

### NSD3L controls rDNA transcription

Given that rDNA transcription is by enlarge the main biological process occurring in the nucleolus, we then addressed whether NSD3L might be functionally relevant for rRNA transcription. We thus transduced cells with two specific NSD3L shRNAs (alongside a non-targeting control). Cells were then pulsed with Ethynyl-Uridine (EU), and we assayed the signal corresponding to nascent RNA transcripts using the Click-iT assay, as a proxy for newly synthesized rRNA [38]. NSD3L knock-down in HeLa cells significantly decreased nascent RNA fluorescence (Figs. 1E, F, S1K). Similar results were obtained in U-2 OS cells, while NSD3L downregulation on immortalized non-transformed Bj-T human fibroblasts did not impact nascent transcription inside nucleoli (Fig. 1E middle and lower panels, Figs. 1F, S1L, M).

To further corroborate the role of NSD3L on modulating rDNA transcription, we analyzed the levels of the 47S neo-transcribed rRNA precursor by Real Time PCR, taking advantage of a primer pair recognizing 5’-ETS (External Transcribed Spacer), a spacer sequence that is rapidly removed from the newly transcribed pre-rRNA and reflects the transcription rate of rRNAs [39]. In agreement with the Click-iT experiment, we observed that NSD3L depletion strongly reduced 5’-ETS levels specifically in HeLa and U-2 OS cancer cell lines. In line with the previous results, we did not observe any change in 5’-ETS in Bj-T fibroblasts (Fig. 1F), as well as in an additional non-transformed cell line, the Human Dermal Fibroblast (HDF) (Fig. S2D).

Of note, NSD3S expression was not altered upon NSD3L knock-down (Fig. 1F). Additionally, NSD3L loss was not associated with cell cycle alterations (Fig. S3A) nor reduction in the total levels of nucleolar proteins (Fig. S3B). To also exclude that the Pol I binding and more broadly the nucleolar phenotype seen after NSD3L KD could be the result of nucleolar stress, we determined the translocation of the nucleolar protein NPM1 from the nucleoli to the nucleoplasm, as a hallmark of nucleolar stress [40]. To this end, we assessed NPM1 cellular localization by immunofluorescence. As reported in Fig. S3C, D, NPM1 signal was largely dispersed to the nucleoplasm under conditions that trigger nucleolar stress (heat-shock at 42 °C and H_2_O_2_). Conversely, NSD3L KD was not associated with a detachment of NPM1 from the nucleoli as measured by nucleolar fluorescence intensity, suggesting that the effects of NSD3L on rRNA synthesis are not related to nucleolar stress.

Taken together, these results reveal that NSD3L localizes inside nucleoli where it sustains rDNA transcription, specifically in cancer cells.

### NSD3L is required for the binding of Pol I to rDNA loci

**Fig. 2:**
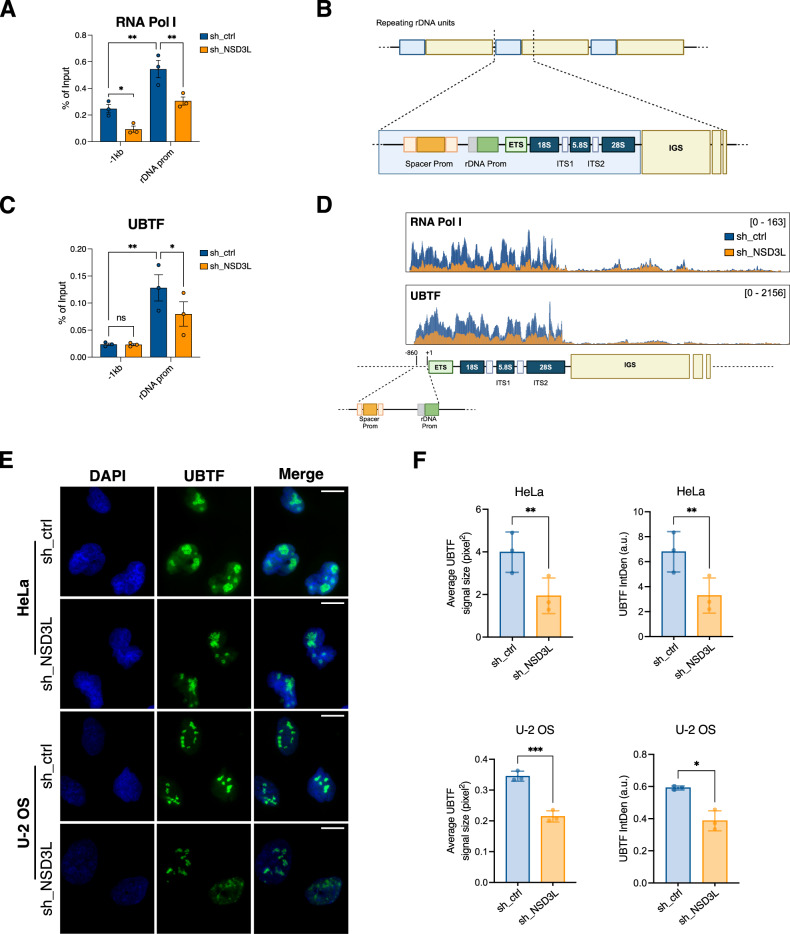
NSD3L knock-down hampers Pol I and UBTF loading on rDNA. **A** RNA Pol I enrichment assessment by ChIP-qPCR upon NSD3L silencing in HeLa cells. Results are expressed as % of input. Data are represented as mean (± SEM) of three independent biological replicates. One-way ANOVA test was performed, with multiple comparison (*= *p* < 0.05, **= *p* < 0.01). **B** Schematic representation of rDNA unit. ETS: External Transcribed Spacer; ITS: Internal Transcribed Spacer; IGS: InterGenic Spacer. **C** UBTF enrichment assessment by ChIP-qPCR upon NSD3L silencing in HeLa cells. Results are expressed as % of input. Data are represented as mean (± SEM) of three independent biological replicates. One-way ANOVA test was performed, with multiple comparison (*= *p* < 0.05, **= *p* < 0.01). **D** Representative IGV tracks of Cut&Tag experiments for Pol I and UBTF on rDNA. Tracks for sh_ctrl (blue) or sh_NSD3L (orange) samples are shown (*n* = 4, from two independent biological replicates). **E** Immunofluorescence in sh_ctrl or NSD3L-silenced HeLa and U-2 OS cells stained for UBTF (green), DDX21 (red) and DAPI (blue) Scale bar: 10 µm. **F** Quantification of average UBTF signal size (pixel^2^) and Integrated Density (IntDen) in sh_ctrl or NSD3L-silenced HeLa and U-2 OS cells. Data are represented as mean (± SD) of three independent biological replicates. Two tails Student’s t-test was performed (*= *p* < 0.05, **= *p* < 0.01, ***= *p* < 0.001).

Seeking to identify the mechanism underlying the activity of NSD3L on rRNA transcription, we first asked whether the binding of Pol I was affected by NSD3L. We hence tested by Chromatin Immunoprecipitation (ChIP) whether NSD3L knock-down impacted on Pol I binding. We detected a significant reduction of Pol I binding at rDNA upon NSD3L KD, as assessed by ChIP (Fig. 2A). Of note, NSD3L knock-down did not reduce total Pol I protein level (Fig. S3B). To gain a more comprehensive perspective on the impact of NSD3L knock-down on Pol I localization on rDNA, we exploited the Cut&Tag technique [41] and assayed cells lacking NSD3L for Pol I binding to rDNA (Fig. 2B). Using the consensus rDNA sequence, we accurately identified Pol I binding regions at rDNA loci (Fig. 2D). NSD3L knock-down dramatically reduced Pol I binding across the entire rDNA sequence, not just at the promoter and TSS regions, suggesting a broad role for NSD3L in recruiting and/or maintaining Pol I at the rDNA locus. While the high similarity of rDNA sequences across loci limits the ability to distinguish the different rDNA locus, we additionally aligned the Cut&Tag reads to the available T2T genome (Fig. S4A-B), providing a confirmation that the reduction in Pol I binding is consistent across these repetitive regions, supporting the robustness of the results obtained with the consensus sequence.

### The localization of the Pol I activator upstream binding factor UBTF is mediated by NSD3L

Provided the role of NSD3L in governing Pol I localization, we assessed whether NSD3L might also impact the binding of upstream proteins known to control the binding of Pol I to rDNA. To this end, we choose UBTF, which not only has a role in recruiting RNA Pol I complex machinery and enhancing rRNA transcription but also in maintaining the Nucleolar Organizing Regions (NoRs) structure [5]. ChIP experiments using the same primers of the Pol I experiments revealed a strong reduction of UBTF binding to rDNA (Fig. 2C), with no change in UBTF total protein levels, upon NSD3L knock-down (Fig. S3B). Again, we relied on Cut&Tag to assess the distribution of UBTF binding to rDNA. As for Pol I, also UBTF bound the entire sequence of rDNA. NSD3L knock-down significantly reduced the binding of UBTF throughout the entire rDNA sequences (Fig. 2D).

To corroborate these observations, we assessed the impact of NSD3L knock-down on UBTF binding within nucleoli by immunofluorescence (Fig. 2E). In both cellular systems, nucleolar UBTF signal was strongly reduced in NSD3L knock-down cells compared to controls as assessed by the quantification of average size and Integrated Density (IntDen) of UBTF signal (Fig. 2F). In HeLa cells, we additionally carried out nucleolar and nucleus-cytoplasmic fractionation followed by Western Blot, as previously reported [42, 43]. Successful separation of nucleoli from nuclei and cytoplasm was verified by the marked depletion of βActin and Lamin-B1 in the nucleolar fraction (Fig. S3E, F). NSD3L knock-down led to a pronounced decrease in UBTF protein levels specifically in the nucleolar compartment, suggesting how NSD3L silencing selectively diminished the nucleolar pool of UBTF without altering its total cellular levels.

Altogether, these results suggest a prominent role for NSD3L as a platform for the localization of the key nucleolar proteins driving rDNA transcription.

### NSD3L genome-wide and rDNA binding sites are shared with AP1 transcription factors

**Fig. 3:**
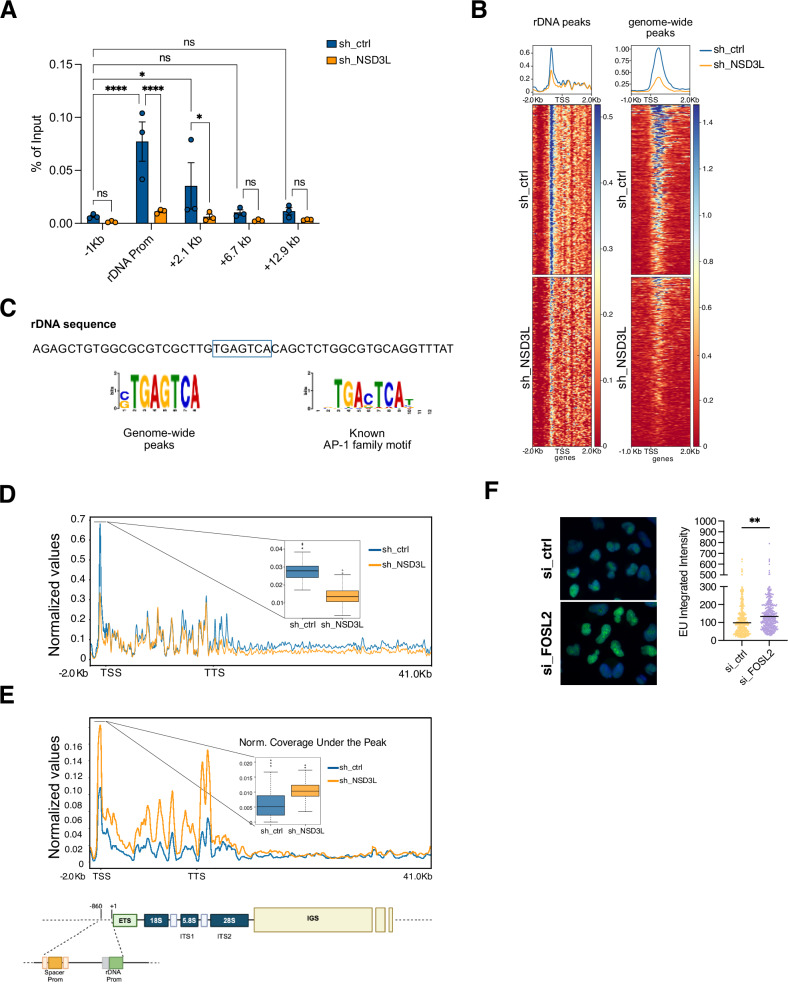
NSD3L directly binds the rDNA promoter region whose sequence is a binding motif also for the AP1 transcriptional factors. **A** NSD3 enrichment assessment by ChIP-qPCR upon NSD3L silencing in HeLa cells. Results are expressed as % of input. Data are represented as mean (± SEM) of three independent biological replicates. A two-way ANOVA test was performed, with multiple comparisons (*= *p* < 0.05, ****= *p* < 0.0001). **B** Heatmap of normalized coverage of NSD3L Cut&Tag signal in sh_ctrl and sh_NSD3L on rDNA (left) and genome-wide (right). The region centered around TSS is shown. The mean score of all regions is shown as a profile above the heatmaps. **C** Novel motif discovery (MEME) across the NSD3L Cut&Tag peaks within the region upstream the TSS of the rRNA genes. Sequences below are extracted from the top 10 known consensus sequences as shown in Supplementary Fig. 5A. **D** Cut&Tag profiles of NSD3L binding on the 43Kb rDNA region represented as the mean of all rDNA clusters from chromosomes 13, 14, 15, 20, 21. In the insert, a boxplot showing the normalized coverage under the NSD3L peak, upstream rDNA TSS (Transcription Start Site). For the average: sh_ctrl and sh_NSD3L (n=4, from two independent biological replicates). TTS: Transcription Termination Site. **E** Cut&Tag profiles of FOSL2 binding on the 43Kb rDNA region represented as the mean of all rDNA clusters from chromosomes 13, 14, 15, 20, 21. In the insert, a boxplot showing the normalized coverage under the FOSL2 peak, upstream rDNA TSS. For the average: sh_ctrl (*n* = 2) and sh_NSD3L (*n* = 5) from two independent biological replicates. **F** Representative images showing EU incorporation followed by Click-iT® assay (left panel) in controls or FOSL2-silenced HeLa cells. Scale bar: 10 µm. On the right panel, quantification of the EU Integrated Intensity of three independent biological replicates. A two-tailed Student’s t-test was performed (**= *p* < 0.001).

Given the nucleolar localization of NSD3L and its role in driving Pol I and UBTF binding to rDNA, we then asked whether NSD3L itself was able to bind rDNA. Using the same ChIP primers used for Pol I and UBTF, we found that NSD3L strongly binds to rDNA in proximity of rDNA promoter (Fig. 3A). Of note, NSD3S was unable to bind to rDNA (Fig. S4C-D). NSD3L bound rDNA also in U-2 OS cells (Fig. S4E) but not in Bj-T immortalized cells (Fig. S4F).

We then expanded our ChIP analysis to additional sequences on rDNA to determine whether NSD3L, as Pol I and UBTF, was able to bind the entire rDNA locus. To our surprise, NSD3L binding was weaker inside the rDNA locus than in the promoter region (Fig. 3A). Prompted by this observation, we comprehensively assayed NSD3 chromatin occupancy with Cut&Tag. This analysis confirmed that, unlike Pol I and UBTF, NSD3L binds upstream of the rDNA locus, specifically on a region between -0.8 and -0.6 Kb from the TSS (Fig. 3B). Intrigued by these results, we hence asked whether this pattern was evident also throughout the genome. We thus identified genome-wide Cut&Tag peaks that were significantly downregulated after knocking-down NSD3L in HeLa cells. We found that outside of rDNA, NSD3L binds instead downstream of the TSS as well as to the first introns and exons of genes (Fig. 3B). In all, these results suggest that on rDNA, NSD3L binds in a specific area upstream of the spacer promoter (Fig. 2B), unlike its genome-wide binding pattern, where NSD3L localizes mostly to TSS and first introns and exons.

Prompted by these results, we then explored whether the regions bound by NSD3L shared any specific consensus sequences. To address this question, we exploited the SEA/MEME database and software [44]. Remarkably, the de novo analysis on the genome-wide NSD3L-specific peaks revealed a consensus motif (TGAC/GTCA), which was also present in the spacer promoter (Fig. 3C). We then sought to determine whether this sequence represented the binding site of known transcription factors (TFs). To this end, we used the SEA database and software [44] for known motifs, and we determined that the TF family recognizing the most similar sequences belonged to the activator protein-1 (AP-1) family members (Fig. S5A). Specifically, FOS, JUN, MAF, and ATF consensus sequences were almost identical to both rDNA NSD3L binding region and the genome-wide peaks (Fig. 3C and S5A-B).

### FOSL2 binds on rDNA locus and represses rDNA transcription

We next sought to obtain conclusive evidence that the AP-1 TF family binds at the rDNA locus, and specifically at the spacer promoter. We leveraged a recently published meta-analysis of TF ChIP-seq assays, where 192 TF profiles have been collected from a large array of 2,200 studies [45]. Strikingly, the ChIP-seq profiles reported for FOS/JUN on the rDNA locus were overlapping with NSD3L binding site displayed by our Cut&Tag results, with the transcription factor FOSL2 (Fra-2) demonstrating the most similar pattern, with a sharp binding pattern on the same region where NSD3L associates with rDNA (Fig. S5C). To confirm these in silico data, we assayed by Cut&Tag FOSL2 DNA binding. We indeed determined that FOS2L bound on the spacer promoter (Fig. 3E).

We next asked whether NSD3L may impact on FOSL2 occupancy on rDNA loci. FOS2L binding to this region and more generally to the entire rDNA locus, robustly increased upon NSD3L loss (Fig. 3E).

Given the role of NSD3L in eliciting ribosomal gene transcription, these results suggest a surprising, opposite, and repressive role for FOSL2 on rDNA transcription. To test this hypothesis, we transiently silenced FOSL2 (Fig. S5D-F). Click-iT RNA imaging assay revealed that FOSL2 knock-down enhanced nascent RNA fluorescence signal (Fig. 3F), implying that FOSL2 may act as a negative regulator of rDNA transcription.

In all, these results suggest that NSD3L and FOSL2 bind preferentially to the rDNA spacer promoter on the rDNA. Additionally, NSD3L loss favors the binding of FOSL2 on the same site, where FOSL2 likely hampers rRNA transcription.

#### The balance between H3K36me2 and H4K20me3 at rDNA regulates ribosomal gene expression

**Fig. 4:**
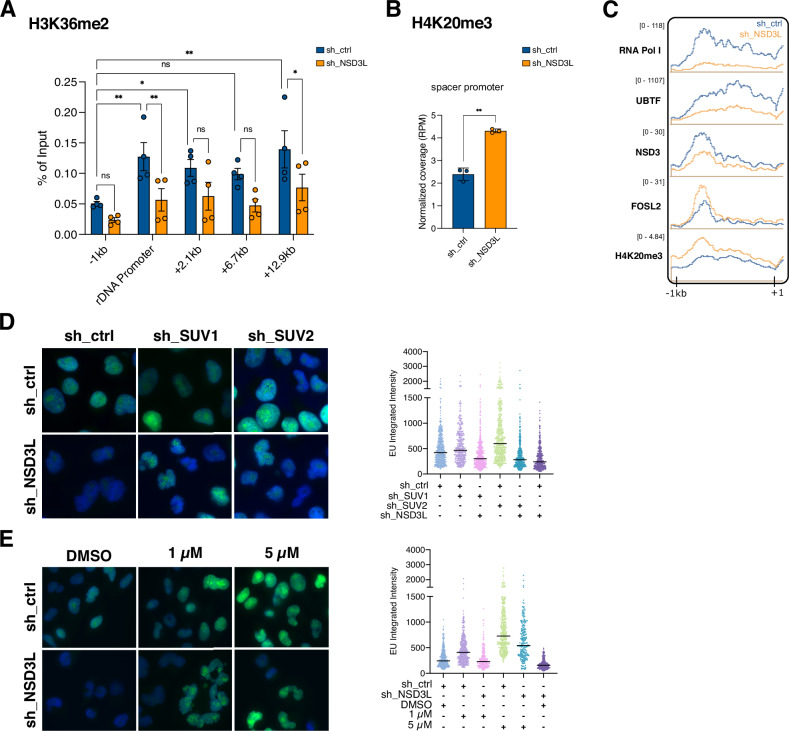
Impact of NSD3L knock-down on rDNA chromatin environment and role of SUV4-20H and H4K20me3. **A** H3K36me2 assessment by ChIP-qPCR upon NSD3L silencing in HeLa cells. Results are expressed as % of the input. Data are represented as mean (± SEM) of four independent biological replicates. A two-way ANOVA test was performed, with multiple comparisons (*= *p* < 0.05, **= *p* < 0.01). **B** Normalized coverage of H4K20me3 on rDNA promoter assessed by Cut&Tag. Data are represented as mean (± SD) of three independent biological replicates. Welch's t-test was performed (**= *p* < 0.01). **C** IGV tracks of RNA Pol I, UBTF, NSD3, FOSL2 and H4K20me3 Cut&Tag experiments. Tracks for sh_ctrl (blue) or sh_NSD3L (orange) samples are shown on the rDNA region between -1Kb and +1Kb. **D** Representative images showing EU incorporation followed by Click-iT® assay (left panel) in controls or silenced HeLa cells using sh_ctrl, sh_NSD3L, sh_SUV1, sh_SUV2 or a combination of them. Scale bar: 10 µm. On the right panel, quantification of EU Integrated Intensity. Statistical details are provided in Supplementary Fig. S7C. **E** Representative images showing EU incorporation followed by Click-iT® assay (left panel) in sh_ctrl or sh_NSD3L HeLa cells and treated with DMSO or 1 μM/5 μM of SUV-420H inhibitor, A196. Scale bar: 10 µm. On the right panel, quantification of EU intensity. Statistical details are provided in Supplementary Figure S7D.

NSD3L adds di- and tri-methyl groups to the histone tail H3K36 [28, 46, 47]. We hence sought to determine whether NSD3L impacts H3K36 methylation on rDNA. NSD3L knock-down strongly reduced H3K36me2 levels throughout the entire rDNA gene body (Fig. 4A), and to a lesser extent H3K36me3 (Fig. S6B), with no changes in H3 levels (Fig. S6A). Also in U-2 OS, but not in Bj-T cells, H3K36 methylation was reduced on the rDNA promoter region upon NSD3L knock-down (Fig. S6C, D).

H4K20me3 has been implicated in chromatin compaction and transcriptional silencing of rDNA [14]. We thus assayed whether NSD3L knock-down may affect H4K20me3 levels at the rDNA promoter. Remarkably, we found a robust increase in the H4K20me3 signal on the spacer promoter, upon NSD3L knock-down (Fig. 4B).

Combining and aligning all the Cut&Tag data (Fig. 4C), we may conclude that NSD3L controls rDNA expression, orchestrating the binding of several key proteins involved in rDNA transcription on the narrow region upstream of the rDNA TSS, as attested by the preferential binding of NSD3L, Pol I, and FOSL2, as well as by the enrichment of the H4K20 repressive histone mark on this narrow genomic region. Accordingly, it seems that the spacer promoter exerts an important role in modulating the activity of the entire rDNA locus and consequently of rRNA transcription, providing the central hub where key proteins modulating rDNA transcription compete for binding.

The enzyme responsible for the deposition of methyl groups on H4K20 is the histone methyltransferase SUV4-20H. We hence explored whether NSD3L could modulate the activity of SUV4-20H at the rDNA. As expected, SUV4-20H genetic knock-down, using two different shRNAs (Fig. S7A), increased nascent RNA fluorescence signal, as determined with the Click-iT RNA imaging assay (Figs. 4D, S7C). The concomitant downregulation of NSD3L (Fig. S7B) prevented the gain in RNA transcription elicited by SUV4-20H down-regulation (Figs. 4D, S7C), suggesting a contrasting effect between NSD3L and SUV4-20H in modulating rDNA expression.

In parallel, we determined whether a selective SUV4-20H inhibitor, namely A-196 [48], could restore the levels of nascent RNA. At two different concentrations [48], again the EU signal increased proportionally to the drug concentration. These effects were significantly hampered upon NSD3L knock-down (Fig. 4E and S7D), suggesting again competitive activities between SUV4-20H and NSD3L, and more broadly between H4K20me3 and H3K36me2 and 3 on the rDNA locus.

In all, these results suggest that NSD3L enhances rDNA transcription in human cancers by loosening the brake around rDNA, counteracting the activity of SUV4-20H, and enhancing transcription initiation. This epigenetic environment, driven by NSD3L, favors the binding of positive regulators (such as Pol I and UBTF) while hampering the binding and activity of factors repressing rDNA transcription, including FOSL2.

### NSD3L expression correlates with genes encoding for nucleolar proteins in human cancers

**Fig. 5:**
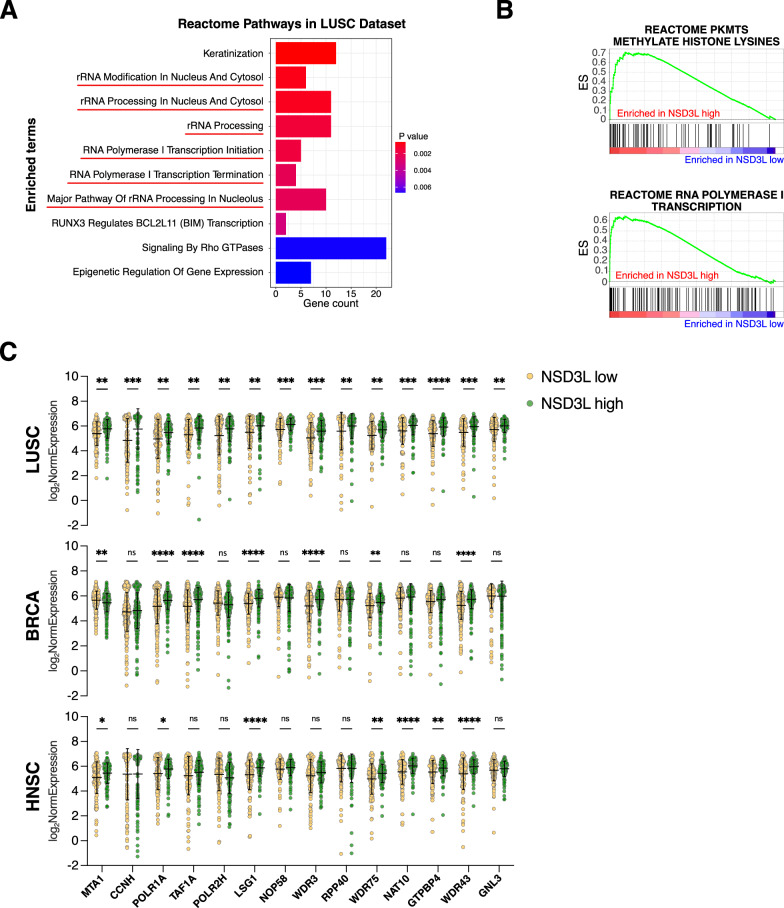
NSD3L expression correlates with genes of key nucleolar pathways in human cancers. **A** Top 10 most enriched (*p*-value) Reactome Pathways by Enrichr of Limma transcriptomic differentially upregulated genes (FDR < 0.05, log2FC > 0) in NSD3L high expression samples (upper quartile) vs low expression samples (lower quartile). **B** Gene Set Enrichment Analysis (v4.3.2) of Reactome Pathways (v2022.1) in LUSC (TCGA pancancer [25]) samples with high expression of NSD3 long isoform (positively correlated) (upper quartile) vs LUSC samples with low expression (negatively correlated) (lower quartile). The GSEA algorithm was applied to the LUSC upper/lower quartiles sample comparison (see methods). **C** Dot-plot with normalized expression levels of selected upregulated genes in NSD3L high expression vs low expression within enriched pathways “Reactome: Polymerase I Transcription Initiation R-HSA-73762" and “KEGG: Ribosome biogenesis in eukaryotes” for the Lung Squamous Carcinoma (LUSC), Breast Invasive Carcinoma (BRCA) tumor types, and Head and Neck Squamous Cell Cancer (HNSC). ES: Enrichment Score.

NSD3 is one of the most frequently amplified genes in cancer, most notably in squamous lung cancer [23, 24]. To determine whether NSD3L impacts on ribosomal gene expression in this cancer type, we explored the TCGA primary tumor datasets (LUSC) [25], seeking to identify pathways more associated with NSD3L overexpression in this cancer type. Most of the significantly overexpressed pathways in patients with higher expression of NSD3L belonged to networks related to ribosomal RNA transcription (Fig. 5A, Supplementary Table S2). This result was confirmed also using Gene Set Enrichment Analysis (GSEA) [49], which revealed ‘RNA Pol I Transcription’ as well as ‘PKMTs methylate histone lysines’, the latter expected of a lysine methyltransferase, as the top Reactome Pathways correlated to high levels of NSD3L (Fig. 5B, Supplementary Table S3). Since NSD3 is amplified in several additional tumor types, we explored whether NSD3L is associated with increased expression of this set of RNA Pol I Transcription and ribosome biogenesis also in other cancer types where NSD3 is frequently amplified, such as head and neck and breast cancers (using the BRCA and HNSC TCGA datasets, respectively [25, 50]). Most of the genes included in the form RNA Pol I Transcription Reactome Pathway and Ribosome biogenesis KEGG pathway, upregulated in LUSC patients with higher levels of NSD3L isoform, also showed enhanced levels of expression in the BRCA and HNSC patient samples endowed with NSD3L high expression, such as POL1RA, WDR43, MTA1 and WDR75 (Fig. 5C and Supplementary Tables S4 and S5, respectively). Altogether, these results indicate that in cancer types where NSD3L is amplified and overexpressed, NSD3L strongly correlates with the expression of several key nucleolar genes, sparking rRNA transcription and ribosome biogenesis.

## Discussion

**Fig. 6:**
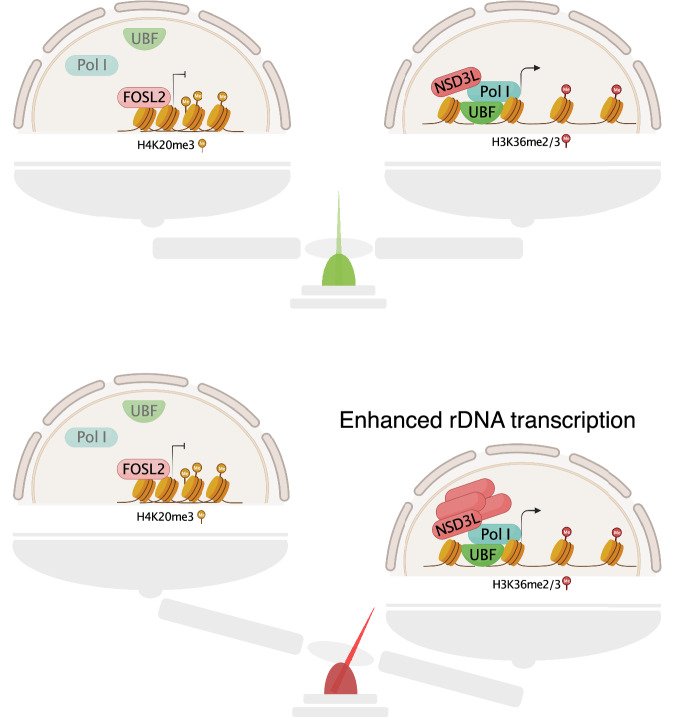
Proposed model for the role of NSD3 long isoform on rDNA regulation in healthy and NSD3-amplified cancer cells. In healthy cells, the long isoform of NSD3 competes with SUV4-20H and FOSL2, contributing to maintain a balance between activation and repression of rDNA transcription (upper panel). In cancers harboring NSD3 amplification (bottom panel), the repressive effects of both SUV4-20H and FOSL2 are lost. As a result, rDNA transcription is enhanced, leading to uncontrolled ribosome biogenesis that supports growth and proliferation of cancer cells.

Our study identifies the long isoform of the histone methyltransferase NSD3 (NSD3L) as a critical epigenetic regulator of rDNA transcription in cancer. We show that NSD3L localizes to the nucleolus, where it engages several nucleolar proteins and binds a specific region upstream of the rDNA transcription start site within the spacer promoter. This focal binding appears to create a permissive chromatin environment that enables the recruitment and maintenance of UBTF and RNA polymerase I across the entire rDNA repeat, thereby sustaining high levels of rRNA transcription (Fig. 6). Our data suggest that NSD3L exerts its function through deposition of H3K36me2 and H3K36me3 marks, which are typically associated with active chromatin, while simultaneously counteracting the repressive activity of SUV4-20H and its associated H4K20me3 mark. Loss of NSD3L leads to a sharp decrease in H3K36 methylation and a concomitant increase in H4K20me3 at the spacer promoter, revealing an epigenetic switch that controls rDNA transcription. Unexpectedly, we also found that FOSL2, a member of the AP-1 transcription factor family, binds the same spacer promoter region and acts as a negative regulator of rDNA transcription when NSD3L is absent. AP-1 plays an important role in various human diseases, most prominently in cancer [51, 52]. However, there are only scattered references for a potential role of AP-1 in ribosome biogenesis [53, 54]. Here, we demonstrated that FOSL2, in the absence of NSD3L, represses rDNA transcription, prompting the idea of a suppressive function for this complex, specifically on ribosome biogenesis (Fig. 6). These results are intriguing, as FOSL2 has always been shown to be a powerful activator of transcription [55]. Indeed, AP-1 proteins, firstly identified as viral oncoproteins [56, 57], have an established oncogenic role. However, JUN and FOS proteins are also tumor suppressors, in specific settings. For instance, whereas c-FOS, FOSB, and c-JUN are tumorigenic when overexpressed in cells and in mice models [58], JUNB and JUND can have anti-oncogenic effects as shown in rodent fibroblasts [59] and in in vivo models of myeloid leukemia [60]. Collectively, whether AP-1 is oncogenic or anti-oncogenic depends on the context, tumor type, tumor stage, and the genetic background [61]. Accordingly, our findings suggest that FOSL2 may have tumor suppressive functions specifically on ribosome biogenesis, revealing a novel mechanism by which cancer cells upregulate ribosome production to support rapid growth.

Ribosome biogenesis has been considered for a long time just as a bystander actor in oncogenesis. Accruing evidence suggests a more active, direct role, as several oncogenes induce cancer directly through the ignition of rDNA transcription [3, 62]. Notwithstanding, very little is known about how cancer cells trigger and sustain the activation of this machinery. Our findings position NSD3L among the very few oncogenes that directly activate Pol I–dependent transcription, a process previously attributed mainly to MYC [18] and provide a mechanistic explanation for the enhanced ribosome biogenesis observed in tumors harboring NSD3 amplification. It is also tempting to speculate that NSD3L may represent the ultimate conduit exploited by other oncogenes to elicit ribosome biogenesis, as suggested by the cancer-specific ability of NSD3L to drive rDNA transcription specifically in the cancer cell lines HeLa and U-2 OS cells (where the 8p11-12 locus is not amplified per se) but not in healthy human fibroblasts (Bj-T and HDF). As a matter of fact, it is conceivable that NSD3L may interact and cooperate with MYC itself to drive ribosome biogenesis, creating a permissive chromatin environment on rDNA, as for example NSD3S, that prevents MYC degradation by FBXW7 [63]. Furthermore, our observations expand the current understanding of rDNA regulation, which has historically focused on repressive complexes such as NoRC and NuRD [13], and only rarely on activators, such as HMT G9a. G9a is localized at active rDNA genes, forming a complex with Cockayne syndrome group B (CSB) and Pol I [64]. G9A triggers the di-methylation of H3K9, leading to the recruitment of the heterochromatin protein family-1 gamma (HP1γ) and facilitating Pol I transcription. Accordingly, knock-down of G9a prevents the loading of Pol I into rDNA [64]. How a repressive mark like H3K9me3 may cooperate with H3K36 methylation, per se a histone modification associated with gene transcription activation, remains to be seen. The role of methylation of lysine H3K36 on histone H3 is multifaceted. H3K36me3 is distributed across the intragenic region of actively transcribed genes and is associated with active chromatin. H3K36me2 is mostly found on active promoters and enhancers [65]. H3K36 methylation has also been involved in transcriptional activation [66]. Limited information is available on the role of H3K36 methylation within rDNA. In mammalian cells, the lysine demethylase KDM2A represses rDNA transcription by demethylating H3K36me2 under glucose starvation [67]. It is then reasonable to speculate that NSD3L is the histone methyltransferase that deposits this histone mark in rDNA, thus maintaining rDNA transcription, opposing the effect of KDM2A. The methylation of histone H4, mostly detected on lysine 20 (H4K20), is associated with many physiological processes, including heterochromatin formation, cell cycle regulation, DNA damage repair, and recombination [68]. H4K20me3 is typically associated with silenced heterochromatin, and it is found near chromosome centromeres and telomeres [68, 69]. Human cancer cells frequently undergo an overall loss of H4K20me3, especially on repetitive regions, and this is associated with malignant transformation [70]. Additionally, the role of H4K20me3 has been described at rDNA clusters by Bierhoff et al. [14]. Indeed, they suggested that trimethylation of H4K20 establishes a chromatin compaction that throttles rRNA synthesis upon growth factor deprivation, providing an explanation for why H4K20me3 is often lost in human cancers [14]. Our data suggest that NSD3L overexpression may be an additional mechanism by which H4K20me3, on rDNA and elsewhere in the genome, may be erased or at least counteracted in cancer cells.

We were surprised that the binding of UBTF, a key factor in the regulation of rDNA transcription, was impacted by NSD3L throughout the entire rDNA locus. It has been held for a long time that UBTF localizes specifically to the rDNA promoter, where it contributes to the pre-initiation complex formation through the recruitment of SL1 and Pol I [5, 7]. ChIP-seq studies have challenged this view, since UBTF seems instead to be enriched throughout the entire rDNA repeat, and minimally to the IGS [5]. Besides confirming this distribution, our Cut&Tag experiments revealed that UBTF binding was almost completely abrogated over the entire rDNA sequence, upon NSD3L knock-down. Given the preferential localization of NSD3L on the spacer promoter, it is tempting to speculate that the binding to this region by NSD3L prompts the focal recruitment of UBTF, which then spreads throughout the entire rDNA region, possibly mediated by H3K36 methylation. To finely tune the binding of UBTF, Pol I, NSD3, FOSL2, and histone marks to rDNA, we leveraged the recently published complete human genome sequence, the so-called T2T (telomer to telomere) [2] sequence. This new data is shedding light on regions of the genome that, so far, have remained obscure and largely excluded from genomic and transcriptomic analyses, such as all centromeric satellite arrays, recent segmental duplications, and the short arms of all five acrocentric chromosomes, which include the complex ribosomal gene arrays. However, the repetitive nature of rDNA still limits precise locus-specific analysis.

In all, these findings have important therapeutic implications. Ribosomal DNA transcription is becoming an attractive entry point for cancer therapeutic efforts [71, 72]. For example, the RNA Pol I transcription factor SL-1 has been successfully targeted with a small-molecule inhibitor in preclinical models [73]. Targeting rDNA transcription may be effective for treating a vast array of cancers. Unlike the short isoform, the long form NSD3L presents the SET catalytic domain, which may be amenable to therapeutic inhibition itself [74–76]. The 8p11-12 amplification is a frequent occurrence in cancer. Hence, we envision that interfering with NSD3L enzymatic activity may represent an enticing strategy to target tumor cells in cancer patients harboring NSD3L amplification and overexpression.

## Method details

### Cell culture

HeLa and U-2 OS cells were purchased from American Type Culture Collection (ATCC) and cultured in Dulbecco’s modified Eagle’s medium (DMEM) (EuroClone) supplemented with 10% fetal bovine serum (EuroClone) and penicillin–streptomycin antibiotics (Merck). Normal human immortalized fibroblast Bj-T (kindly provided by Fanciulli’s Lab, Regina Elena Hospital, Rome) was cultured in a mix of DMEM (EuroClone) (1/4 v/v), F199 (Sigma) (3/4) (supplemented with 15% fetal bovine serum, 1X non-essential amino acids (Invitrogen), and penicillin-streptomycin antibiotics. HeLa and U-2 OS cell lines were authenticated using the short tandem repeat profiling service of Eurofins Genomics (Ebersberg, Germany), and all cells were routinely tested to exclude mycoplasma contamination.

### Transfection

For overexpression and silencing experiments, 3 × 10^5^ cells were plated the day before transfection to obtain 70% confluency the day of transfection. Transfections to overexpress NSD3L were performed using FuGENE® HD Transfection Reagent (Promega) with a FuGENE: DNA ratio of 3:1. Cells were collected for RNA extraction 48 hours after transfection. To silence FOSL2, we used MISSION^®^ esiRNA (Merck) specific for FOSL2 (cat. no. EHU072911) or MISSION^®^ esiRNA targeting a control RLUC region (cat. no. EHURLUC) as a control. HeLa cells were transfected using Oligofectamine Reagent (Thermofisher Scientific) with esiRNAs at a final concentration of 30 nM according to the manufacturer’s instructions. FOSL2 mRNA, protein levels, and biological effects were assessed 24 h post-transfection.

### Lentiviral production and transduction

Four specific shRNAs targeting NSD3L and SUV4-20H, together with a non-targeting shRNA, referred as sh_ctrl, were purchased from Merck and then cloned into pLKO.1 lentiviral vector through a double digestion with AgeI and EcoRI restriction enzymes. shRNAs are then ligated into linearized vectors. The hairpin sequences were cloned into pLKO.1 vectors are provided in Table 1 (Supplementary material).

To obtain lentiviral particles for the infections, HEK-293T cells were transfected with the CaCl_2_ method. All the cell lines used were incubated overnight with a mix 1:2 of lentivirus-containing supernatant/cell culture medium. The following day, cells were detached in Trypsin-EDTA and cultured in antibiotic-containing medium (puromycin was used at 2 µg/mL for U-2 OS and Bj-T; 1.5 µg/mL for HeLa cells and 1 µg/mL for HDF cell lines, for 72 h). Thus, for each independent experiment, the infection protocol was performed in a timeframe of 5 days.

### RNA extraction, reverse transcription, and Real Time PCR

Total RNA was extracted using the RNeasy® Mini Kit (Qiagen) from 5×10^5^ cells, following the manufacturer’s instructions. The ImProm-IITM Reverse Transcription System (Promega) was used to synthesize cDNA with random primers. Quantitative Real Time PCR was performed using SYBR® Green Master Mix (Applied Biosystems) on the ViiA™ 7 Real Time PCR System (Applied Biosystems). The output was normalized on the expression of GAPDH and analyzed with the ΔCt or ΔΔCt methods. Data are represented as mean (±SD) of three independent experiments. A list of the primers used for the different Real Time PCR experiments is provided in Table 2 (Supplementary material).

### Western blot

Protein samples obtained from 10^6^ cells were resuspended in Laemmli Sample Buffer 1X (50 mM Tris-HCl pH 6.8, 2% SDS, 10% glycerol, 100 mM dithiothreitol, 0.01% bromophenol blue), sonicated with a Bioruptor^®^ UCD-200 TO (Diagenode) sonicator (10% amplitude, 3 pulses of 15 seconds each), and heat-denatured (10 minutes at 95°C). Proteins were electrophoretically separated with precast NuPAGE^®^ Novex^®^ 4–12% Bis-Tris Gels (Invitrogen) and transferred onto nitrocellulose membrane (Amersham Hybond ECL, GE Healthcare) with a BioRad Trans-Blot^®^ system. Membranes were incubated with primary antibodies listed in Table 3 (Supplementary material). Secondary HRP-conjugated antibodies used in this study: α-mouse, α-rabbit (both from GE Healthcare, 1:10,000) and α-goat (Santa Cruz Biotechnology, 1:10 000). For immunoprecipitation experiments, TrueBlot® ULTRA HRP α-rabbit and α-mouse secondary antibodies were used (eBioscience, 1:5 000). ECL Detection System (GE Healthcare) was used for the chemiluminescent reaction. A list of antibodies used is provided in Table 3 (Supplementary material).

### Mass spectrometry

Total input and immunoprecipitated lysates were separated on NuPAGE® Novex® 4–12% Bis-Tris Gel (Invitrogen) and stained with Coomassie Blue. Gel regions excluding heavy and light immunoglobulin chains were processed as described in [77]. Samples were analyzed via nano-scale liquid chromatography tandem mass spectrometry (nLC-MS/MS) on a ThermoFisher Orbitrap mass

spectrometer coupled to Easy 1000 nanoLC (ThermoFisher). Data were analyzed using Mascot and X! Tandem with the uniprot_cp_hum_2012_02 database.

### Protein immunoprecipitation

Each experiment was performed with at least 1,5 × 10^7^ cells. All protein purifications were carried out on cell nuclei prepared by 20 min swelling in nuclear prep buffer (10 mM Tris-HCl pH 7.6, 100 mM NaCl, 2 mM MgCl_2_, 0.3 M Sucrose, 0.25% v/v Igepal) at 4 °C. After 8 minutes of incubation on ice followed by a 10 minutes, 8000 x *g* at 4 °C centrifugation, nuclei were lysed in high salt buffer (20 mM Tris–HCl pH 7.5, 300 mM NaCl, 10% glycerol, 0.25% Igepal) with fresh addition of a protease inhibitor cocktail (Roche). The lysate was sonicated 4 cycles (10 seconds on/40 seconds off) with a probe sonicator (Bandelin Sonifier). A total of 250 U of DNAse I and 5 mM MgCl_2_ were added to 1 mg DNA equivalent. DNA was estimated by measuring UV absorption at 260 nm in a 2 M NaCl solution. Lysates were then incubated for 1 hour on ice and centrifuged 1 hour at max speed, at 4 °C. The supernatant was divided in two aliquots and supplemented with specific antibody or isotypic IgG as a control. Samples were placed on the rotation wheel overnight, at 4 °C, after addition of 50–70 μL of Dynabeads protein G (Invitrogen) pre-washed with lysis buffer. 2% of the supernatant was resuspended in Laemmli 1X (Total Input). The mixture was then washed 4/5 times with Lysis Buffer and finally eluted with Laemmli 2X. The eluted supernatant represents the immunoprecipitate (IP). Samples were then boiled at 95° for 5 minutes and then analyzed by WB.

### Immunofluorescence

HeLa and U-2 OS cells were fixed in ice-cold methanol for 10 min at RT. Cells were then permeabilized with 0.3% Triton X-100 (Sigma) in PBS for 10 min at RT. After three PBS washes, a specific binding site was blocked by treatment with blocking solution (PBS/3% Bovine Serum Albumin (BSA)) for 30 min at RT. Staining with primary antibodies was carried out for 1 h at RT. After three PBS washes, cells were then incubated with the secondary Alexa-488 and Alexa-546 antibodies for 1 h RT (at a dilution of 1:1000 in 3%BSA/PBS). Coverslips were mounted on glass slides with ProLong® Gold Antifade Reagent with 4’,6-diamidino-2-phenylindole (DAPI) (Invitrogen). For confocal microscopy, a Leica Confocal – TCS SP5 Laser Scanning Confocal at the ALEMBIC (Advanced Light and Electron Microscopy Bio-Imaging Centre, San Raffaele Scientific Institute) facility was used. Primary antibodies were used at a dilution of 1:1000 for NSD3, 1:250 for UBTF and DDX21 staining, and 1:100 for NPM1. Where indicated, images from EU experiments were analyzed using Cell Profiler pipelines [78], and images from immunofluorescence experiments were quantified using ImageJ [79]. A minimum of 20 cells per field was analyzed.

### Click-iT® RNA Imaging Assay

Click-iT® RNA Imaging Assay (Promega) was performed according to the manufacturer’s instructions. Briefly, cells were plated on coverslips and transfected as indicated, and 48 hours later were pulsed with 1 mM 5-ethynyl uridine (EU) in complete DMEM for 15 minutes. Cells were then washed, fixed in 4% PFA, and permeabilized with 0.5% Triton® X-100 for 15 minutes. Staining was performed with Click-iT® reaction mixture containing the azide-conjugated Alexafluor® 488 dye for 30 minutes at RT. DNA staining was performed with Hoechst 33342 diluted 1:1000 in PBS, for 15 minutes. Mounting was performed using ProLong® Gold Antifade Reagent (Invitrogen), and images of cells were collected using fluorescence microscopy. Data analysis was performed taking advantage of a pipeline developed in the open-source software Cell Profiler. Briefly, cells were automatically identified, and all nuclei signal intensity was measured. GraphPad Prism was used for statistical analysis and data visualization.

### Proximity Ligation Assay (PLA)

HeLa and U2-OS cells were seeded at 50% confluence on coverslips in 12-well plates. After 24 hours, cells were fixed with 4% PFA for 10 min at RT and permeabilized with 0.3% Triton-X for 2 h, and then PLA was performed following the customer’s instructions (PLA Duolink Merck). Briefly, cells were incubated with the primary antibodies against UBTF (1:2500 dilution, SantaCruz Biotech.) and NSD3 (1:500 dilution, Proteintech) overnight at 4° C. Then, incubation with the probes was performed. After ligation and amplification, slides were mounted by using in situ mounting media with DAPI and acquired at 100X magnification by Axio Imager.A2 fluorescence microscope (Zeiss).

ImageJ [79] was used to process each image. The PLA puncta were reliably isolated from the background and then counted. Quantification of nuclei proceeds similarly. The result was expressed as PLA puncta/nuclei. Significant differences among groups were calculated by applying an unpaired Student t-test.

### Chromatin immunoprecipitation

Chromatin was collected and purified as in [80]. Lysates were sonicated with Bioruptor (Diagenode) to obtain products ranging from 300 to 500 bp. Lysates from HeLa and U-2 OS cells were sonicated 10-12 cycles (30” ON, 30” OFF, high intensity). 50–100 μg of ready-to-use chromatin were used for IP with anti-NSD3, anti-UBTF, anti-Pol I, while 10–20 μg were used for each IP to histones. For each ChIP, Dynabeads protein G slurry (Invitrogen) was incubated with 6-8 μg antibody (3 μg antibody for ChIP on histones). Samples were purified with the QIAquick PCR purification kit (Qiagen), following the manufacturer’s recommendations. DNA was eluted in TE buffer, and Real Time PCR Sybr^®^ Green Real Time PCR kit (Invitrogen) was performed. Three biological replicates for each ChIP experiment are reported as mean ± SEM. Data are reported as a percentage of input normalized to H3. Primer sequences were obtained from [81], and are listed in Table 4 (Supplementary material). Primers are named according to their position relative to TSS, which is referred as *position 0*.

### Cleavage under targets and tagmentation (Cut&Tag)

Cut&Tag assays were performed as described previously [41]. Nuclei from HeLa cells were extracted with NE1 buffer (Hepes-KOH 1 M, pH7.9; KCl 1 M; Spermidine 2 M; Triton X-100 10%; glycerol; protease inhibitors; H_2_O) and cryopreserved in 10% DMSO and wash buffer (Hepes 1 M, pH 7.5; NaCl 5 M; Spermidine 2 M; protease inhibitors; H_2_O). Frozen aliquots of nuclei were thawed, dispensed in low-retention PCR tubes, and bound to Concavalin A magnetic beads (Bangs Laboratories, BP531). For NSD3, FOSL2, UBTF, and Pol I experiments, 8 × 104 nuclei were lightly cross-linked (0,1% formaldehyde) for 2 minutes and blocked by addition of 1.25 M glycine. Samples were incubated O/N with the primary antibodies listed in Table 3 (Supplementary material). The guinea pig anti-Rabbit (Antibodies online, ABIN101961) or the rabbit anti-Mouse (Abcam, ab46540) secondary antibodies were incubated in 1:100 dilution for 30 minutes. Protein A/G–Tn5 (pAG-Tn5) fusion protein loaded with double-stranded adapters (Epicypher, 15-1117) was mixed with the bead-bound nuclei at RT for 1 hour. Tagmentation was allowed for 1 hour at 37° C then, particles were released at 58 ° C for 1 hour. Cut&Tag libraries were prepared with dual index oligos [82] and the amplification was performed with NEBNext 2X PCR Master mix (New England BioLabs, ME541L). Library purification was applied using HighPrep PCR Cleanup (Magbio Genomics, AC-60500) and paired-end sequenced through NovaSeq 6000 SP (Illumina, San Diego, CA, USA).

### Alignment to the T2T genome

Cut&Tag reads were aligned to the human CHM13 T2T genome, containing the most accurate to date assembly and description of rDNA human clusters [2]. Since human rDNA arrays represent the most complex and repetitive region in the T2T genome, we used a variation of the strategy used by the consortium to map protein binding to the centromeres [83]. After trimming of adapters with trimmomatic [84], reads were aligned using Bowtie 2 with -k 100 as a parameter, allowing for the reporting up to 100 mapped loci for each read and hence producing overfitted profiles that constitute an upper bound limit for enrichment. This modality was chosen over the single-locus k-mer filtered procedure due to the highly repetitive nature of the rDNA locus [2]. Alignments were then processed as above. For differential peak calling, DiffBind [73] was used for pre-processing and consensus peak definition and counting (peaks present in at least 2 out of 4 replicates were retained), and DESeq2 for statistical analysis (RLE normalization, pValue < 0.01). For rDNA and genome-wide building of Cut&Tag profiles and heatmaps, deeptools2 was used. Briefly, normalised bigwigs were obtained from filtered alignments using the bamCoverage function with BPM (Bins per Million Reads) normalization, ignoring duplicates and in 10 bp bins. Profiles and heatmaps were built using computeMatrix and plotProfile/plotHeatmap functions using bigWigs to scale coverage around TSS of both genome-wide genes with bona-fide NSD3L peaks (downregulated in shNSD3L compared to shCTRL, pValue < 0.01) or TSS of rDNA genes in T2T annotation for averaged track profiles (using wiggletools *mean* function) (For NSD3: sh_ctrl (*n* = 4) and sh_NSD3L (*n* = 4); For FOSL2: sh_ctrl (*n* = 2) and sh_NSD3L (*n* = 5)). IGV 2.12.3 was used for Cut&Tag coverage track visualization using hg38 and the T2T CH13Mv2.0 genome.

### TCGA data analysis

RSEM normalised isoform-level data matrices for Lung Squamous Cell Carcinoma (LUSC), Breast Invasive Carcinoma (BRCA) and Head and Neck Squamous Carcinoma (HNSC) were downloaded from https://gdac.broadinstitute.org/runs/stddata__2016_01_28/data servers. The levels of the NSD3L isoform (ENST00000317025, WHSC1L1-NSD3-202 in the original annotation nomenclature) were used to define two groups of patients: high (normalized levels of expression above the third quartile (0.75) and low expression (normalized levels of expression below the first quartile (0.25). Then, standard differential gene expression analysis using LIMMA [85] was applied (FDR < 0.05) on gene-level TCGA data matrices to find genes upregulated in the NSD3L high expression group compared to low expression. *Enrichr* package [37] was used to check for functionally enriched pathways and Gene Ontology (Reactome, KEGG, BioPlanet, and GO Cellular Component). In parallel, gene-level expression matrices were used to test the correlation of NSD3L high vs low phenotype with gene expression by Gene Set Enrichment Analysis (v4.3.2) against Reactome Pathways. DotPlots were made with GraphPad Prism 9.0, using log2Normalized gene-level expression of genes upregulated both in the LUSC dataset for the enriched Reactome Pathway ‘RNA Pol I Transcription’ and KEGG Pathway ‘Ribosome biogenesis’, for the three indicated datasets. Additional significance of the compared levels of expression was tested using an unpaired t-test.

### Motif-based consensus sequence analysis

The de-novo consensus sequence (MEME) and enriched sequence analysis (SEA) under rDNA and genome-wide NSD3L peaks were performed using the online MEME-suite tools (MEME, SEA) with default parameters [44].

### Statistical analyses

The number of biological replicates (n) was chosen based on standard practice and reported in each figure legend. Statistical analyses were performed using GraphPad Prism 10. Results were displayed as mean ± standard error of the mean (SEM) or standard deviation (SD) of a minimum of three independent biological replicates. Significant differences among groups were quantified by applying Student’s *t*-test or one-way ANOVA, followed by post hoc multiple comparison tests, as specified in each figure legend. A significance was assumed at *p*-values *p* < 0.05. No randomization or blinding was applied as experiments involved cultured cell lines under controlled conditions.

## Supplementary information

Supplementary material

Supplementary material

## Data Availability

Cut&Tag data has been deposited in the Gene Expression Omnibus database. Data are registered as GSE224372 (The histone methyltransferase NSD3 triggers ribosomal DNA transcription interfering with FOSL2 in cancer).
