# Supplementary material for: The histone methyltransferase NSD3 oncogene triggers ribosomal DNA transcription, interfering with FOSL2 in cancer
Source: Cell Death Dis. 2026 May 4;17(1):592. doi: 10.1038/s41419-026-08768-0 (PMC13287619; doi:10.1038/s41419-026-08768-0)
Supplement: Supplementary file 1 — Supplementary material [file 41419_2026_8768_MOESM1_ESM.docx]

**Supplementary material**

**FIGURE LEGENDS**

**Fig. 1 - NSD3L interacts with nucleolar proteins and localizes in the nucleolus**

**(A)** Top 10 enriched pathways using GO Biological Processes 2021 or **(B)** Cellular Component 2021 using Enrichr for NSD3L interactors identified with mass spectrometry. **(C)** Confocal microscopy on HeLa, U-2 OS and Bj-T cells, expressing the N-terminal GFP-NSD3L fusion protein and stained with an anti-UBTF nucleolar marker. Scale bar: 7 µm. **(D)** Proximity Ligation Assay (PLA) foci quantified as PLA puncta/nuclei in HeLa and U-2OS cells sh_ctrl and sh_NSD3L conditions. Significant differences among groups were quantified by applying Student’s t-test (**** p<0.0001). Scale bar: 10 µm. **(E)** Representative images showing EU incorporation followed by Click-iT® assay (scale bar: 10 µm) on the left. On the right, quantification of EU signal intensity expressed as mean integrated intensity (IntDen). Data are represented as mean (± SD) of three independent biological replicates. One-way ANOVA test was performed, with multiple comparison (***= p< 0.001, ****= p< 0.0001). **(F)** RNA expression levels of NSD3L, NSD3S and 5’ ETS, assessed with Real Time qPCR on HeLa, U-2 OS, and BJ-T cell lines, silenced with two different shRNAs targeting NSD3L. Data are normalized first on GAPDH then to control condition. Results are represented as mean (± SD) of three independent biological replicates. Where indicated, a two tails Student’s t-test was performed (*** p<0.001, **** p<0.0001).

**Fig. 2 - NSD3L knock-down hampers Pol I and UBTF loading on rDNA**

**(A)** RNA Pol I enrichment assessment by ChIP-qPCR upon NSD3L silencing in HeLa cells. Results are expressed as % of input. Data are represented as mean (± SEM) of three independent biological replicates. One-way ANOVA test was performed, with multiple comparison (*= p< 0.05, **= p< 0.01). **(B)** Schematic representation of rDNA unit. ETS: External Transcribed Spacer; ITS: Internal Transcribed Spacer region; IGS: InterGenic Spacer region. **(C)** UBTF enrichment assessment by ChIP-qPCR upon NSD3L silencing in HeLa cells. Results are expressed as % of input. Data are represented as mean (± SEM) of three independent biological replicates. One-way ANOVA test was performed, with multiple comparison (*= p< 0.05, **= p< 0.01). **(D)** Representative IGV tracks of Cut&Tag experiments for Pol I and UBTF on rDNA. Tracks for sh_ctrl (blue) or sh_NSD3L (orange) samples are shown (n=4, from two independent biological replicates). **(E)** Immunofluorescence in sh_ctrl or NSD3L-silenced HeLa and U-2 OS cells stained for UBTF (green), DDX21 (red) and DAPI (blue) Scale bar: 10 µm. **(F)**  Quantification of average UBTF signal size (pixel^2^) and Integrated Density (IntDen) in sh_ctrl or NSD3L-silenced HeLa and U-2 OS cells. Data are represented as mean (± SD) of three independent biological replicates. Two tails Student’s t-test was performed (*= p< 0.05, **= p< 0.01, ***= p< 0.001).

**Fig. 3 -** **NSD3L directly binds the rDNA promoter region whose sequence is a binding motif also for the AP1 transcriptional factors**

**(A)** NSD3 enrichment assessment by ChIP-qPCR upon NSD3L silencing in HeLa cells. Results are expressed as % of input. Data are represented as mean (± SEM) of three independent biological replicates. Two-way ANOVA test was performed, with multiple comparison (*= p< 0.05, ****= p< 0.0001). **(B)** Heatmap of normalized coverage of NSD3L Cut&Tag signal in sh_ctrl and sh_NSD3L on rDNA (left) and genome-wide (right). The region centered around TSS is shown. The mean score of all regions is shown as a profile above the heatmaps. **(C)** Novel motif discovery (MEME) across the NSD3L Cut&Tag peaks within the region upstream the TSS of the rRNA genes. Sequences below are extracted from the top 10 known consensus sequences as shown in Supplementary Figure 5A. **(D)** Cut&Tag profiles of NSD3L binding on the 43Kb rDNA region represented as the mean of all rDNA clusters from chromosomes 13, 14, 15, 20, 21. In the insert, a boxplot showing the normalized coverage under the NSD3L peak, upstream rDNA TSS (Transcription Start Site). For the average: sh_ctrl and sh_NSD3L (n=4, from two independent biological replicates). TTS: Transcription Termination Site. **(E)** Cut&Tag profiles of FOSL2 binding on the 43Kb rDNA region represented as the mean of all rDNA clusters from chromosomes 13, 14, 15, 20, 21. In the insert, a boxplot showing the normalized coverage under the FOSL2 peak, upstream rDNA TSS. For the average: sh_ctrl (n=2) and sh_NSD3L (n=5) from two independent biological replicates. **(F)** Representative images showing EU incorporation followed by Click-iT® assay (left panel) in controls or FOSL2-silenced HeLa cells. Scale bar: 10 µm. On the right panel, quantification of EU Integrated Intensity of three independent biological replicates. Two tails Student’s t-test was performed (**= p< 0.001).

**Fig. 4 - Impact of** **NSD3L knock-down on rDNA chromatin environment and role of SUV4-20H and H4K20me3**

**(A)** H3K36me2 assessment by ChIP-qPCR upon NSD3L silencing in HeLa cells. Results are expressed as % of input. Data are represented as mean (± SEM) of four independent biological replicates. Two-way ANOVA test was performed, with multiple comparison (*= p< 0.05, **= p< 0.01). **(B)** Normalized coverage of H4K20me3 on rDNA promoter assessed by Cut&Tag. Data are represented as mean (± SD) of three independent biological replicates. Welch's t test was performed (**= p< 0.01). **(C)** IGV tracks of RNA Pol I, UBTF, NSD3, FOSL2 and H4K20me3 Cut&Tag experiments. Tracks for sh_ctrl (blue) or sh_NSD3L (orange) samples are shown on the rDNA region between -1Kb and +1Kb. **(D)** Representative images showing EU incorporation followed by Click-iT® assay (left panel) in controls or silenced HeLa cells using sh_ctrl, sh_NSD3L, sh_SUV1, sh_SUV2 or a combination of them. Scale bar: 10 µm. On the right panel, quantification of EU Integrated Intensity. Statistical details are provided in supplementary figure S7C. **(E)** Representative images showing EU incorporation followed by Click-iT® assay (left panel) in sh_ctrl or sh_NSD3L HeLa cells and treated with DMSO or 1μM/5μM of SUV-420H inhibitor, A196. Scale bar: 10 µm. On the right panel, quantification of EU intensity. Statistical details are provided in supplementary figure S7D.

**Fig. 5 - NSD3L expression correlates with genes of key nucleolar pathways in human cancers
(A)** Top 10 most enriched (p-value) Reactome Pathways by Enrichr of Limma transcriptomic differentially upregulated genes (FDR < 0.05, log2FC > 0) in NSD3L high expression samples (upper quartile) vs low expression samples (lower quartile). **(B)** Gene Set Enrichment Analysis (v4.3.2) of Reactome Pathways (v2022.1) in LUSC (TCGA pancancer (25)) samples with high expression of NSD3 long isoform (positively correlated) (upper quartile) vs LUSC samples with low expression (negatively correlated) (lower quartile). GSEA algorithm was applied to the LUSC upper/lower quartiles sample comparison (see methods). **(C)** Dot-plot with normalized expression levels of selected upregulated genes in NSD3L high expression vs low expression within enriched pathways “Reactome: Polymerase I Transcription Initiation R-HSA-73762" and “KEGG: Ribosome biogenesis in eukaryotes” for the Lung Squamous Carcinoma (LUSC), Breast Invasive Carcinoma (BRCA) tumor types and Head and Neck Squamous Cell Cancer (HNSC). ES: Enrichment Score.

**Fig. 6 - Proposed model for the role of NSD3 long isoform on rDNA regulation in healthy and NSD3-amplified cancer cells**

In healthy cells, the long isoform of NSD3 competes with SUV4-20H and FOSL2, contributing to maintain a balance between activation and repression of rDNA transcription (upper panel). In cancers harboring NSD3 amplification (bottom panel), the repressive effects of both SUV4-20H and FOSL2 are lost. As a result, rDNA transcription is enhanced, leading to uncontrolled ribosome biogenesis that supports growth and proliferation of cancer cells.

**Supplementary Figures**

**Fig. S1 - NSD3L interacts with key nucleolar proteins.**

**(A)** WB analysis of NSD3L protein levels in HeLa overexpressed with GFP_NSD3L construct or H1703 and JIMT-1 cell lines. Actin protein levels were used as loading control. **(B)** Densitometric analysis of the WB experiment shown in panel A. **(C)** Flag-NSD3L was immunoprecipitated with an anti-Flag antibody or an anti-normal IgG. IP was verified through WB using anti-NSD3 antibody. TI: Total Input. **(D)** SDS PAGE of immunoprecipitated proteins stained with InstantBlue^TM^ (Sigma-Aldrich) with the indicated antibodies. **(E)** IP performed on endogenous NSD3 protein using an anti-NSD3 antibody or anti-normal IgG as a control in HeLa or **(F)** U-2 OS cells. **(G)** IP performed in HeLa cells expressing HA-NSD3S or LacZ control, using an anti HA-antibody, in the presence of RNAse A. Total Input (TI) is shown as non-immunoprecipitated control in all gels. **(H)** Additional representative immunofluorescence images of UBTF (red) or DAPI (blue) in HeLa cells overexpressed with GFP_NSD3L (Scale bar: 10 µm). **(I)** Proximity Ligation Assay (PLA) negative controls on HeLa cells showing no dots when only probes or only antibodies (Abs) are stained. **(J)** Confocal microscopy images of HeLa, U-2 OS and Bj-T cells, expressing HA-tagged NSD3 short (NSD3S) and stained with an anti-DDX21 nucleolar marker. Scale bar: 15 µm. **(K-L-M)** Representative images, from Click-iT assays of Figure 1E, showing single channels for EU, DAPI and Merge in HeLa (K), U-2 OS (L) and Bj-T (M) cell lines.

**Fig. S2 - NSD3L interactors belong to nucleolus-related pathways and processes.**

**(A)** Top 10 GO Biological Processes 2021 enriched using EnrichR, ordered by Fisher p-value with statistics. (Combined score = -log10pValue * Odds Ratio). **(B)** Top 10 enriched pathways using KEGG Human 2021 (left panel) with statistics (right panel). **(C)** List of top 10 cellular compartments using GO Cellular Components 2021, with statistics. **(D)** RNA expression levels of NSD3L and 5’-

ETS, assessed with Real Time qPCR on HDF cell line, normalized to GAPDH and then compared controls versus sh_NSD3L samples. Data are represented as mean (± SD) of five independent biological replicates. A two tails Student’s t-test was performed (**** p<0.0001).

**Fig. S3 - NSD3L knock-down does not alter key nucleolar protein levels.**

**(A)** Cell cycle analysis of HeLa and U2-OS control cells compared to NSD3L silenced cells, using propidium iodide (PI) staining, and flow cytometry. Data are represented as mean (± SD) of three independent biological replicates. Two-way ANOVA test was performed with Tukey’s multiple comparison. All differences were not statistically significant (ns). **(B)** WB analysis of NSD3L-interacting proteins upon NSD3L knock-down using two different shRNAs. ACTIN protein levels were used as loading control. **(C)** Representative immunofluorescence images of the nucleolar NPM1 (red) or DAPI (blue) in HeLa or U-2 OS cells in controls or sh_NSD3L. As positive controls, samples were heat-shocked at 42°C (30 min) or exposed to H_2_O_2_ (500µM for 30 minutes). **(D)** Measurement of nuclear Fluorescence Intensity (FI), in all conditions were performed with ImageJ(1). Significant differences among groups were calculated by one way ANOVA followed by Tukey’s post-test for multiple comparison (*= p< 0.05, **= p< 0.01, ***= p< 0.001, ****= p< 0.0001). Scale bar: 5 µm. **(E)** Representative WB analysis of UBTF protein levels upon NSD3L knock-down in the nucleolar compartment (Nu) and in the cytoplasm + nuclei (C+N). LAMINB1 and ßACTIN were used as a nuclear or cytoplasmic specific positive control, respectively. NPM1 was loaded as nucleolar positive control. **(F)** Densitometric analysis of three independent biological replicates of WB experiments after nucleolar fractionation. UBTF was normalized on NPM1 level. Student’s t-test was performed (* p<0.05).

**Fig. S4 - NSD3L, but not NSD3S, binds to the rDNA promoter region**

**(A-B)** CUT&Tag profiles of RNA Pol I and UBTF binding, respectively, on the 43Kb rDNA region. Data are represented as the mean of all rDNA clusters on chromosomes 13, 14, 15, 20, 21. Sequences were aligned using the T2T genome. **(C)** ChIP-qPCR experiments performed on HA-NSD3S or **(D)** Flag-NSD3L expressing HeLa cells with anti-HA or anti-Flag antibodies, respectively. The CCND1 (known NSD3S target) exon 2 was applied as a positive control in the HA-NSD3S experiment. Data are represented as mean (± SD) of three independent biological replicates. **(E)** ChIP-qPCR experiments performed on U-2 OS cell line or **(F)** Bj-T cells, performed with an anti-NSD3 antibody. Results are expressed as % of input normalized on -1Kb control region. Data are represented as mean (± SEM) of three independent biological replicates. One tail, paired t-test was performed (*= p< 0.05).

**Fig. S5 - NSD3L binding sites are shared with AP1 transcriptional factors**

**(A)** Simple Enrichment Analysis (SEA) of known consensus sequences in genome-wide NSD3L bound regions (Cut&Tag peaks significantly downregulated after NSD3L knock-down). Top 10 enriched consensus sequences are shown. **(B)** Novel motif discovery (MEME), in the same regions described in A. **(C)** Representative ChIP-Seq profile of an AP-1 family transcription factor, FOSL2, from *Anthony et al., 2022*, on the rDNA locus. **(D)** RNA expression levels of FOSL2 in HeLa control or FOSL2-silenced cells, normalized on GAPDH. Data are represented as mean (± SD) of three independent biological replicates. A two tails Student’s t-test was performed (** p<0.01). **(E)** WB analysis of FOSL2 silencing with esiRNA. Vinculin was used as loading control. **(F)** Densitometric analysis of three independent biological replicates of WB experiments. A two tails Student’s t-test was performed (** p<0.01).

**Fig. S6 - NSD3L knock-down reduces H3K36me2 and me3 levels**

**(A)** H3 ChIP enrichment levels on rDNA promoter and coding region in HeLa cells upon NSD3L knock-down. Data are represented as mean (± SD) of three independent biological replicates. **(B)** ChIP-qPCR experiments performed in control (sh_ctrl) or NSD3L silenced (sh_NSD3L) HeLa cells, U-2 OS cells **(C)** or **(D)** Bj-T cells using antibodies recognizing H3K36me2/me3 as indicated. Results are expressed as % of input. Data are represented as mean (± SEM) of three independent biological replicates. Paired t-test, one-tailed was performed (*= p< 0.05, ****= p< 0.0001).

**Fig. S7 - The balance between H3K36me2 and H4K20me3 at rDNA regulates ribosomal gene expression (A)** RNA expression levels of SUV4-20H and NSD3L **(B)** in HeLa cells upon SUV-420H or NSD3L knock-down, normalized on GAPDH and on sh_ctrl. Data are represented as mean (± SD). Two tails Student’s test was performed compared to sh_ctrl (*= p< 0.05, **= p< 0.01, ***= p< 0.001, ****= p< 0.0001). **(C)** Tables containing the statistical analyses from figure 4D and **(D)** figure 4E. Two tails Mann-Whitney test was performed on three independent biological replicates.

**Tables**

| **shRNAs** | **Sequence** |
| --- | --- |
| sh_ctrl | CCGG**CAACAAGATGAAGAGCACCAA**CTCGAG**TTGGTGCTC TTCATCTTGTTG**TTTTTG |
| sh1_NSD3L | CCGG**AACAGAAGAGACGAAAGATCA**CTCGAG**TGATCTTTCGTCTCTTCTGTT**TTTTTG |
| sh2_NSD3L | CCGG**ATTAGTTACACTGTTCTCGTG**CTCGAG**CACGAGAACAGTGTAACTAAT**TTTTTG |
| sh_SUV1 | CCGG**GCACCCATAAGATGAACGTCA**CTCGAG**TGACGTTCATCTTATGG GTGC**TTTTTG |
| sh_SUV2 | CCGG**GTGTCCACTCGTGCTTGGAAA**CTCGAG**TTTCCAAGCACGAGTGG ACAC**TTTTTG |

**Table 1. List of the hairpin sequences cloned in pLKO.1 vectors.** siRNA sense and antisense sequences are highlighted.

| **Primers** | **Forward** | **Reverse** |
| --- | --- | --- |
| NSD3L | AAAAGGAGCATTAAGAAGGGTG | GTTACACTGTTCTCCTGGGCTC |
| NSD3S | GTTGTGCCAAAGAAGAAGATCA | CTGACAGAGCCCTGCACTC |
| 5'-ETS | CACGTGTTTCCTCGTACC | ACGCCTCTCAGATCGCTAGA |
| FOSL2 | CAGAAATTCCGGGTAGATATGCC | GGTATGGGTTGGACATGGAGG |
| GAPDH | AGCCACATCGCTCAGACAC | GCCCAATACGACCAAATCC |

**Table 2. List of** **primers used for the Real Time PCR experiments.**

| **Target Protein** | **Species** | **Company** | **Applications** |
| --- | --- | --- | --- |
| NSD3 | Rabbit | Proteintech | WB, IP, ChIP, IF, Cut&Tag, PLA |
| H3 | Rabbit | Abcam | WB, ChIP |
| H3K36me2 | Rabbit | Abcam | WB,ChIP |
| H3K36me3 | Rabbit | Abcam | WB, ChIP |
| H4K20me3 | Rabbit | Merck | WB, ChIP |
| Normal mouse IgG | Mouse | Santa Cruz | WB,ChIP |
| Normal rabbit IgG | Rabbit | Santa Cruz | WB, ChIP |
| RPA135 (Pol I) | Goat | Santa Cruz | WB, ChIP, IP, Cut&Tag |
| UBTF [F-9] | Mouse | Santa Cruz | WB, ChIP, IF, IP, Cut&Tag, PLA |
| actin-HRP | Mouse | BD Biosciences | WB |
| DDX21 | Rabbit | Novus Biologicals | WB, IF |
| NPM1 [na24] | Mouse | Thermofisher | WB, IF |
| FOSL2 | Rabbit | CST | WB, Cut&Tag |
| FLAG [M2] | Mouse | Merck | IP, WB |

**Table 3. List of** **antibodies used for WB, IP, ChIP, Cut&Tag, IF and PLA experiments.**

| **Primers** | **Forward** | **Reverse** |
| --- | --- | --- |
| -1 Kb | CCGTGGGTTGTCTTCTGACT | AAGCGAAACCGTGAGTCG |
| rDNA prom | CTGCGATGGTGGGCGTTTTTG | ACAGCGTGTCAGCATATAACC |
| +2.1 Kb | GACCGCCCTCGTGTCTGT | GGGGGAAGAAGAGGATCG |
| +6.7 Kb | GCAGGACACATTGATCATCG | GACGCTCAGACAGGCGTAG |
| +12.9 Kb | ACCTGGCGCTAAACCATTCGT | GGACAAACCCTTGTGTCGAGG |

**Table 4. List of** **primer sequences used for ChIP experiments.**

**Supplementary Tables**

**Table S1**. **List of NSD3 co-immunoprecipitating proteins identified by mass spectrometry.**

The number of unique peptides relative to each hit are reported for IgG and IP lanes.

**Table S2. List of pathway enrichment results of LUSC dataset**

Results of pathway enrichment using EnrichR of LUSC dataset using Reactome 2022, BioPlanet 2019, KEGG 2021 Human, GO Cellular Component 2021, with statistics. Combined score: -log10(Fisher p-value) * Odds Ratio.

**Table S3. List of GSEA results**

Top 20 Reactome Pathways correlated with high levels of NSD3L.

**Table S4. List of pathway enrichment results of BRCA dataset**

Results of pathway enrichment using EnrichR of BRCA dataset using Reactome 2022, BioPlanet 2019, KEGG 2021 Human, GO Cellular Component 2021, with statistics. Combined score: -log10(Fisher P-value) * Odds Ratio.

**Table S5. List of pathway enrichment results of HNSC dataset**

Results of pathway enrichment using EnrichR of HNSC dataset using Reactome 2022, BioPlanet 2019, KEGG 2021 Human, GO Cellular Component 2021, with statistics. Combined score: -log10(Fisher P-value) * Odds Ratio.

1. J. Schindelin, *et al.*, Fiji: an open-source platform for biological-image analysis. *Nat Methods* **9**, 676–682 (2012).
